# Exploring the Prognostic Role of Red Blood Cell Distribution Width in Aortic Valve Calcification Evaluations via Multi-Slice Computed Tomography

**DOI:** 10.31083/j.rcm2512437

**Published:** 2024-12-12

**Authors:** Yiyao Zeng, Fulu Jin, Li Wang, Peiyu Wang, Hui Xiong, Yafeng Zhou, Yufeng Jiang, Liangping Zhao

**Affiliations:** ^1^Department of Cardiology, The Fourth Hospital Affiliated to Soochow University, Medical Center of Soochow University, Suzhou Dushu Lake Hospital, 215000 Suzhou, Jiangsu, China; ^2^Department of Cardiology, The Second Affiliated Hospital of Soochow University, 215000 Suzhou, Jiangsu, China; ^3^Emergency Department of Xuguan District, The Second Affiliated Hospital of Soochow University, 215000 Suzhou, Jiangsu, China

**Keywords:** red blood cell distribution width, aortic valve calcification, prediction

## Abstract

**Background:**

Previous reports have indicated an association between red blood cell distribution width (RDW) and cardiovascular disease. However, few relevant studies exist on the relationship between RDW and aortic valve calcification (AVC). Explore the correlation and predictive value of RDW concerning the occurrence and severity of aortic valve calcification.

**Methods:**

Blood examination results were analyzed from 1720 hospitalized patients at the Second Affiliated Hospital of Soochow University. Logistic regression analysis and the Cox proportional hazards model examined the relationship between RDW and the incidence and severity of AVC.

**Results:**

The RDW value in cases with AVC was significantly higher than in the control group. Red cell distribution width-standard deviation (RDW–SD) and red cell distribution width-coefficient of variation (RDW–CV) increased with calcification severity. Both RDW–SD and RDW–CV demonstrated high predictive values for the occurrence of aortic valve calcification.

**Conclusions:**

Red blood cell distribution width significantly correlated with the occurrence and severity of aortic valve calcification.

## 1. Introduction

Calcified aortic valve disease (CAVD) is characterized by progressive calcification of aortic leaflet fibers, resulting in deformities, impaired valve function, left ventricular outflow stenosis, and hemodynamic complications [1]. CAVD exhibits a protracted progression, ranging from initial calcified nodules or focal leaflet thickening to eventual severe heart failure [2]. In the context of a globally aging population, CAVD has emerged as a prominent cause of aortic stenosis (AS) in developed nations, ranking as the third largest cardiovascular disease following coronary atherosclerosis and hypertension.

Epidemiologically, CAVD affects approximately 0.4% of the general population, with a prevalence of 1.7% in individuals aged over 65 years. Alarmingly, less than one-third of patients with severe aortic stenosis survive beyond five years [3, 4, 5]. Shu S *et al*. [6] employed an age–period–cohort model to analyze global trends in CAVD between 1990 and 2019, revealing unsatisfactory outcomes with 127,000 deaths attributed to CAVD in this period. Research indicates significant variability in CAVD mortality across different countries. Mortality rates decreased notably in countries with a high sociodemographic index (SDI) [–1.45%, 95% confidence interval (CI) (–1.61 to –1.30)], slightly increased in high-middle SDI countries [0.22%, 95% CI (0.06–0.37)], and remained unchanged in other SDI quintiles [6]. The pathogenesis of CAVD is intricate, involving factors such as elevated circulatory resistance, abnormal valve tension, chronic inflammatory response, extracellular matrix (ECM) remodeling, metabolic disorders, and neovascularization [7]. In contrast to historical perspectives that considered CAVD a passive calcium deposition process associated with aging, contemporary understanding recognizes it as an actively regulated outcome influenced by factors such as inflammation and metabolism.

Red blood cells (RBCs), nucleated blood elements with a distinctive oval double concave shape, play a vital role in oxygen and carbon dioxide transport throughout the body. The red blood cell count is commonly used to assess the severity of anemia and diagnose conditions such as polycythemia vera and congenital hemoglobin disorders, including thalassemia [8]. Red cell distribution width (RDW) indicates the size variability in circulating erythrocytes, expressed as the coefficient of variation of red cell size. Elevated RDW values indicate increased variation in volume differences between red blood cells, often observed in patients with malnutrition or deficiencies in folate or B vitamins [9]. This occurs due to the accelerated degradation of RBCs, leading to the premature release of immature RBCs into the bloodstream, resulting in increased RDW and varying RBC sizes. Indeed, RDW, expressed as red cell distribution width–standard deviation (RDW–SD) and red cell distribution width–coefficient of variation (RDW–CV), is commonly used in diagnosing hematological diseases.

Recent studies have illuminated the strong link between RDW and various cardiovascular diseases, such as heart failure, acute myocardial infarction, and atrial fibrillation, along with other physiological abnormalities, such as peripheral artery disease, chronic kidney disease, chronic obstructive pulmonary disease, sepsis, acute pancreatitis, gastrointestinal disease, and cancer [7, 10]. Despite these findings, the correlation between RDW and aortic valve calcification remains elusive. Thus, this study aimed to elucidate the relationship between RDW and aortic valve calcification by gathering data from patients undergoing multislice computed tomography (MSCT) to assess the presence of aortic valve calcification and its calcification score. RDW may be a rapid and easily assessed biomarker in almost all health facilities. Furthermore, RDW can be obtained as a part of a complete blood count for evaluating the severity and prognosis of patients with CVDs; further investigations are needed to assess the efficacy and accuracy of RDW in CVDs. Our objective was to investigate the connection between RDW and aortic valve calcification and evaluate the predictive significance of using RDW in relation to aortic valve calcification.

## 2. Methods and Materials

### 2.1 Study Population

A total of 1720 hospitalized patients who underwent blood examinations at the Second Affiliated Hospital of Soochow University between 2010 and 2019 were included in this study, following approval from the Medical Ethics Committee of the hospital (Ethics number: 240016).

#### 2.1.1 Inclusion Criteria

Patients meeting the following criteria were included:

The presence of unexplained chest pain and chest tightness, coupled with clinical assessment indicating suspicion of aortic valve stenosis, along with abnormal findings on exercise electrocardiography.

Patients who completed the blood test successfully and also underwent MSCT.

#### 2.1.2 Exclusion Criteria

Patients meeting any of the following criteria were excluded:

Severe general conditions such as renal and hepatic insufficiency, malignancy, and infectious and hemorrhagic diseases. Severe renal insufficiency was defined as an estimated glomerular filtration rate (eGFR) less than 30 mL/min/1.73 m^2^. Liver insufficiency was defined as alanine aminase elevation due to any liver disease that exceeded twice the normal upper limit. Hemorrhagic diseases encompass anomalies in coagulation, platelet function, and vascular wall integrity arising from diverse etiologies.

Patients with unsuccessful MSCT scans.

Inpatients discharged or deceased within 24 hours.

### 2.2 Research Methods

#### 2.2.1 Grouping of Cases

**Fig. 1.**
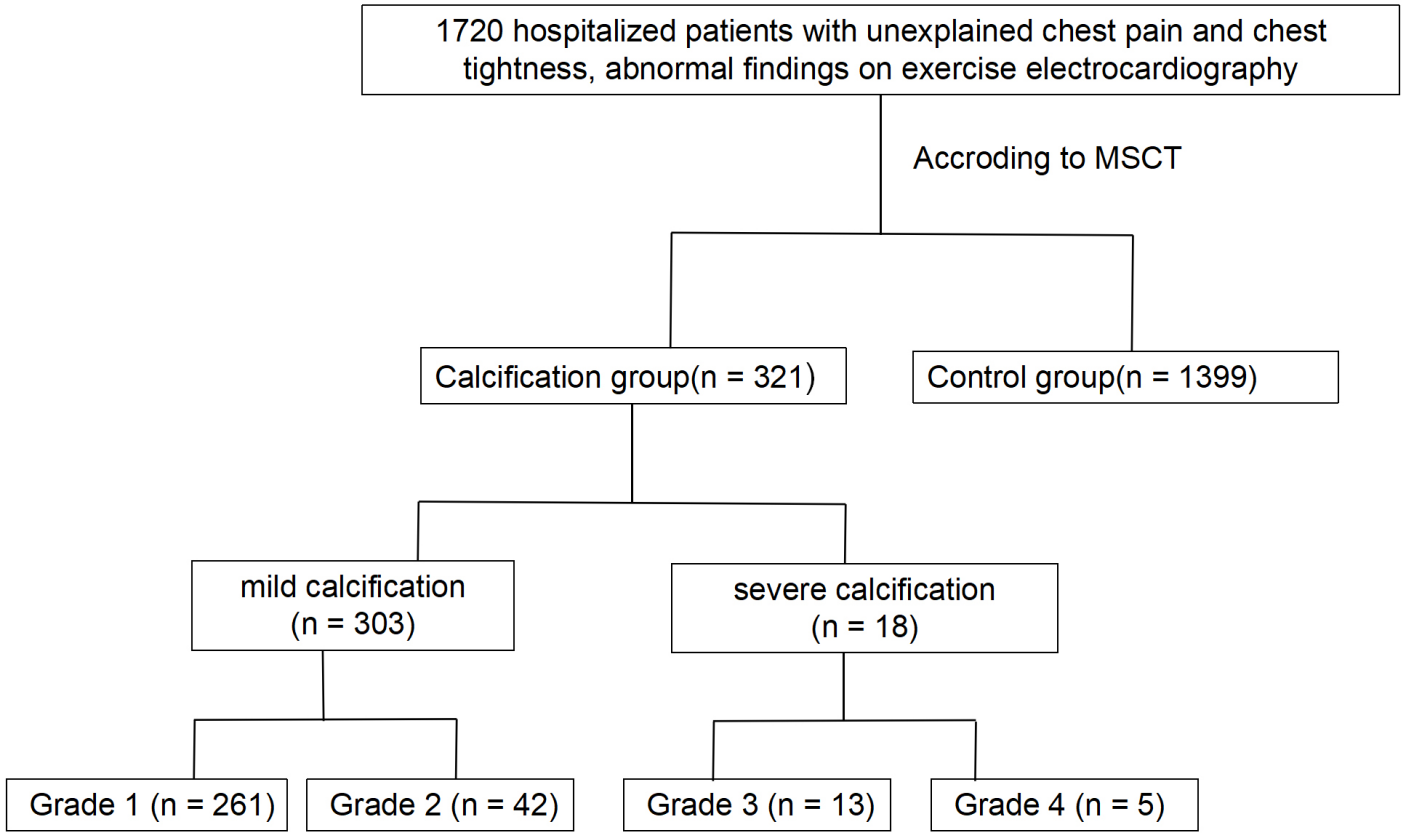
**Design of the study**. MSCT, multislice computed tomography.

The 1720 patients were categorized based on the presence or absence of aortic valve calcification and the corresponding calcification integral. Results showed 321 patients classified in the calcified group (CAVD) and 1399 patients in the control group—those without CAVD (Fig. 1).

#### 2.2.2 General Data Collection

The following demographic and clinical parameters were recorded for the selected patients: age, gender, blood pressure, height, weight, body mass index (BMI), smoking habits, and alcohol consumption.

#### 2.2.3 Concomitant Diseases

Hypertension diagnosis was established based on the criteria of systolic blood pressure ≥140 mmHg or diastolic blood pressure ≥90 mmHg measured at rest. Patients with a documented history of hypertension or current use of antihypertensive medications were considered hypertensive [11].

Diabetes was diagnosed through the presence of diabetes-related symptoms accompanied by a random blood glucose level of ≥11.1 mmol/L, a blood glucose level ≥11.1 mmol/L measured 2 hours after glucose loading, or a fasting blood glucose level ≥7.0 mmol/L [12]. Individuals with a clear history of diabetes mellitus or the use of hypoglycemic medications or subcutaneous insulin injections were also classified as having diabetes mellitus.

The documentation of any previous occurrence of stroke in the patient’s medical history was meticulously recorded.

#### 2.2.4 Aortic Valve Computed Tomography (CT) Examination

Evaluation of Aortic Valve Calcification

The presence and severity of aortic valve calcification were assessed using MSCT. The assessment categorized aortic valve calcification (AVC) into different grades, as previously described [13]:

Grade 1: No calcification observed

Grade 2: Mild calcification, characterized by small, isolated points

Grade 3: Moderate calcification involving multiple large spots

**Fig. 2.**
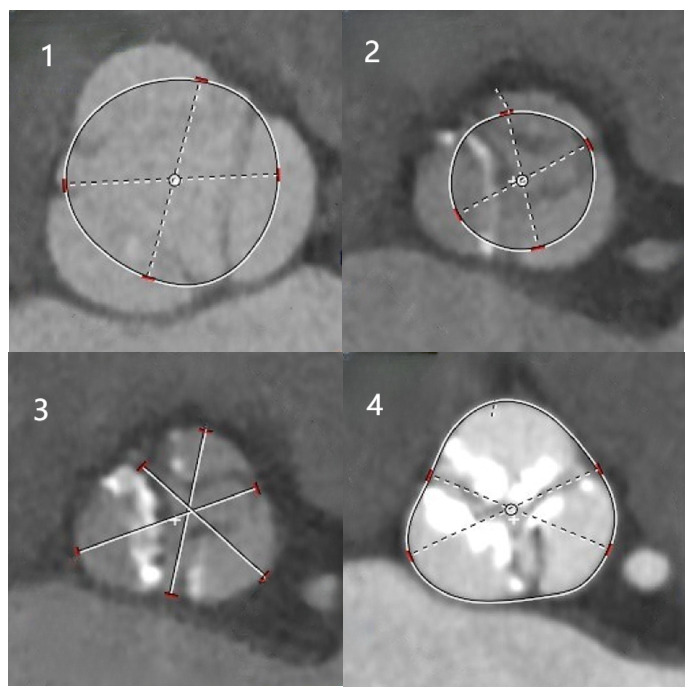
**Aortic valve calcification score**. (1) No calcification; (2) mild calcification; (3) moderate calcification; (4) severe calcification.

Grade 4: Severe calcification, indicated by extensive calcification across all areas of the valve leaflets (Fig. 2).

#### 2.2.5 Coronary Artery Computed Tomography Angiography (CTA) Examination

A 64-slice spiral computed tomography scan was employed for the coronary examination. The coronary artery calcification score (CACS), calculated by Agatston, encompassed the right coronary (RCA), left main (LM), left circumflex branch (LCX), and left anterior descending branch (LAD).

#### 2.2.6 Echocardiography Examination

Complete echocardiography was conducted during hospitalization, capturing the following parameters: left ventricular end-diastolic inner diameter (LVEDD), left ventricular ejection fraction (LVEF), left ventricular end-systolic diameter (LVESD), etc.

#### 2.2.7 Biochemical Examination

Venous blood was obtained upon admission and preserved using ethylenediaminetetraacetic acid (EDTA) anticoagulant. An automated blood cell counter (HmX AL Blood Analyzer, Beckman Coulter, Brea, CA, USA) was employed to evaluate red blood cell counts, hemoglobin, erythrocyte, RDW–SD, and RDW–CV. Standard methods were utilized for assessing leukocytes, platelets, C-reactive protein (CRP), blood-urea-nitrogen, etc. The eGFR was calculated using the Modification of Diet in Renal Disease (MDRD) formula.

#### 2.2.8 Statistical Analysis

For normally distributed data, an independent sample *t*-test was conducted for continuous variables, while the Kruskal–Wallis test was employed for non-normally distributed data. Group comparisons were conducted utilizing the Student–Newman–Keuls (SNK) test across groups. Categorical variables are expressed as the frequency (percentage), and inter-group comparisons were performed using the chi-square test. Multivariate logistic regression analysis was used to initially explore potential influencing factors for the presence of AVC and severe AVC and identify independent influencing factors and their efficacy. The association between elevated RDW–SD, RDW–CV, and AVC was explored using the interquartile range. All data analyses were performed using Windows SPSS 17.0 (IBM Corp., Chicago, IL, USA) , and statistical significance was set at a two-sided *p*-value < 0.05.

## 3. Results 

### 3.1 Comparative Analysis of Basic Clinical Data

In this study involving 1720 patients, 321 had aortic valve calcification (calcification group), while 1399 showed no calcification (control group). The mean age in the calcification group (72.8 ± 8.0 years) was significantly higher than the control group (61.1 ± 11.6 years, *p* < 0.05).

The prevalence of hypertension and diabetes was significantly higher in the calcification group (*p* < 0.05). Patients with calcification had higher systolic blood pressure (144.1 ± 22.2 vs. 137.8 ± 20.1 mmHg, *p* < 0.05) and lower diastolic blood pressure (77.3 ± 12.2 vs. 80.0 ± 12.4 mmHg, *p* < 0.05) compared to controls. Height and weight were significantly lower in the calcification group (*p* < 0.05). No significant variance was observed in the body mass index among the groups (Table 1).

**Table 1.** **Comparison of the clinical characteristics between the calcification group and control groups**.

Characteristics		Control group	Calcification group	*p*-value
		(n = 1399)	(n = 321)	
Basic information				
	Males (n, %)	735 (52.5)	160 (49.8)	0.384
	Age (year)	61.1 ± 11.6	72.8 ± 8.0	<0.001
	Smokers (n, %)	326 (23.3)	67 (20.9)	0.350
	Hypertension (n, %)	803 (57.4)	244 (76.0)	<0.001
	Diabetes mellitus (n, %)	229 (16.4)	85 (26.5)	<0.001
	Systolic blood pressure (mmHg)	137.8 ± 20.1	144.1 ± 22.2	<0.001
	Diastolic blood pressure (mmHg)	80.0 ± 12.4	77.3 ± 12.2	<0.001
	Height (m)	1.64 ± 0.09	1.61 ± 0.08	0.001
	Weight (kg)	66.7 ± 11.9	64.8 ± 11.3	0.009
	Body mass index (kg/m^2^)	24.79 ± 3.52	24.82 ± 3.59	0.909
Blood test				
	White blood cells (10^9^/L)	6.37 ± 1.95	6.35 ± 2.04	0.848
	Red blood cells (10^12^/L)	4.49 ± 0.51	4.23 ± 0.57	<0.001
	Hemoglobin	135.4 ± 15.9	129.9 ± 17.4	<0.001
	Erythrocyte	40.7 ± 4.3	39.1 ± 4.9	<0.001
	RDW–SD	42.16 ± 3.43	43.37 ± 4.20	<0.001
	RDW–CV	12.72 ± 0.92	12.97 ± 1.12	<0.001
	Platelet (10^9^/L)	205.8 ± 68.3	187.9 ± 60.1	<0.001
	PDW	14.8 ± 2.5	14.9 ± 2.5	0.518
	Mean platelet volume	10.7 ± 1.4	10.8 ± 1.6	0.262
	Fasting blood glucose (mmol/L)	5.63 ± 1.64	5.86 ± 2.10	0.037
	Total cholesterol (mmol/L)	4.59 ± 1.02	4.44 ± 1.15	0.026
	Triglyceride (mmol/L)	1.69 ± 1.32	1.60 ± 1.17	0.230
	LDL-C (mmol/L)	2.77 ± 0.85	2.66 ± 0.99	0.068
	HDL-C (mmol/L)	1.17 ± 0.34	1.16 ± 0.36	0.649
	Blood-urea-nitrogen (mmol/L)	5.25 ± 1.62	5.78 ± 2.56	<0.001
	Blood creatinine (µmol/L)	67.8 ± 20.5	71.6 ± 19.0	0.002
	Blood uric acid (µmol/L)	337.9 ± 98.7	347.8 ± 102.3	0.112
	eGFR (mL/min/1.73 m^2^)	96.1 ± 22.2	86.7 ± 22.5	<0.001
	Blood calcium (mmol/L)	2.22 ± 0.26	2.23 ± 0.22	0.569
	Serum inorganic phosphorus (mmol/L)	1.16 ± 0.19	1.15 ± 0.20	0.400
	C-reactive protein (mg/L)	5.40 (4.80–6.40)	7.28 (6.65–9.93)	0.045
	LVEF (%)	65.9 ± 8.1	64.3 ± 9.2	0.003
	LVEDD (mm)	48.3 ± 5.2	49.1 ± 5.8	0.015
	LVESD (mm)	30.8 ± 5.6	31.8 ± 6.5	0.005
	Left atrial internal diameter (mm)	40.3 ± 5.8	43.4 ± 6.6	<0.001
	Interventricular septum (mm)	9.6 ± 1.7	10.0 ± 1.7	0.001
	Left posterior wall (mm)	9.2 ± 1.2	9.5 ± 1.2	<0.001
	Aortic root internal diameter (mm)	32.7 ± 3.8	33.1 ± 3.7	0.113
	Pulmonary artery systolic pressure (mmHg)	28.1 ± 7.2	31.4 ± 9.4	<0.001
	Total calcification score	98.1 ± 368.6	340.2 ± 737.5	<0.001
	LM score	49.1 ± 89.9	90.8 ± 153.4	0.031
	LAD score	125.7 ± 255.4	219.7 ± 277.8	<0.001
	LCX score	61.7 ± 126.8	106.8 ± 170.1	0.009
	RCA score	135.9 ± 371.8	206.3 ± 353.2	0.036
Severity of coronary calcification (n, %)				<0.001
	Low coronary artery calcification (<100 points)	426 (64.4)	103 (41.4)	
	Middle coronary artery calcification (100–400 points)	155 (23.4)	79 (31.7)	
	High coronary artery calcification (>400 points)	80 (12.1)	67 (26.9)	
Coronary artery calcification (n, %)		661 (47.2)	249 (77.6)	<0.001
LM calcification (n, %)		150 (10.7)	76 (23.7)	<0.001
LAD calcification (n, %)		507 (36.2)	208 (64.8)	<0.001
LCX calcification (n, %)		270 (19.3)	126 (39.3)	<0.001
RCA calcification (n, %)		361 (25.8)	169 (52.6)	<0.001
LM lesion (n, %)		193 (13.8)	74 (23.1)	<0.001
Degree of LM stenosis (n, %)				0.199
	<25%	131 (67.9)	45 (60.8)	
	25%–50%	38 (19.7)	14 (18.9)	
	50%–75%	19 (9.8)	9 (12.2)	
	≥75%	5 (2.6)	6 (8.1)	
LAD lesion (n, %)		846 (60.5)	270 (84.1)	<0.001
Degree of LAD stenosis (n, %)				<0.001
	<25%	234 (27.7)	48 (17.8)	
	25%–50%	382 (45.2)	112 (41.5)	
	50%–75%	184 (21.7)	82 (30.4)	
	≥75%	46 (5.4)	28 (10.4)	
LCX lesion (n, %)		391 (27.9)	175 (54.5)	<0.001
Degree of LCX stenosis (n, %)				0.033
	<25%	129 (33.0)	53 (30.3)	
	25%–50%	165 (42.2)	61 (34.9)	
	50%–75%	77 (19.7)	42 (24.0)	
	≥75%	20 (5.1)	19 (10.9)	
RCA lesion (n, %)		611 (43.7)	212 (66.0)	<0.001
Degree of RCA stenosis (n, %)				<0.001
	<25%	208 (34.0)	53 (25.0)	
	25%–50%	294 (48.1)	79 (37.3)	
	50%–75%	79 (12.9)	55 (25.9)	
	≥75%	30 (4.9)	25 (11.8)	
Diseased vessel number (n, %)				<0.001
	0	1099 (78.6)	167 (52.0)	
	1	179 (12.8)	81 (25.2)	
	2	74 (5.3)	38 (11.8)	
	3	47 (3.4)	35 (10.9)	

RDW–SD, red blood cell distribution width–standard deviation; RDW–CV, red blood cell distribution width–coefficient of variation; PDW, platelet distribution width; LDL-C, low-density lipoprotein cholesterol; HDL-C, high density lipoprotein cholesterol; eGFR, estimated glomerular filtration rate; LVEF, left ventricular ejection fraction; LVEDD, left ventricular end-diastolic inner diameter; LVESD, left ventricular end-systolic diameter; LM, left main artery; LAD, left anterior descending branch; LCX, left circumflex branch; RCA, right coronary artery.

### 3.2 Comparative Analysis of Blood Indicators

This study identified significant differences in blood indicators between the aortic valve calcification and control groups. Specifically, RDW–SD (43.37 ± 4.20 vs. 42.16 ± 3.43, *p* < 0.001) and RDW–CV (12.97 ± 1.12 vs. 12.72 ± 0.92, *p* < 0.001) were higher in the calcification group.

In the calcification group, fasting blood glucose, blood-urea-nitrogen, blood creatinine, and C-reactive protein levels were significantly elevated compared to the control group (*p* < 0.05). Conversely, red cell count, hemoglobin, hematocrit, platelet count, total cholesterol, and eGFR were significantly lower in the calcification group (*p* < 0.05) (Table 1).

### 3.3 Comparison of Echocardiography Indicators

Cardiac and vascular parameters differed significantly between patients with aortic valve calcification and the control group. Specifically, LVEF values were lower in the calcification group (64.3 ± 9.2 vs. 65.9 ± 8.1%, *p* < 0.05). In the calcified group, LVEDD, LVESD, left atrial diameter, left ventricular posterior wall thickness, septal thickness, and pulmonary artery systolic pressure increased significantly compared to the control group (*p* < 0.05) (Table 1).

### 3.4 Integration and Distribution of Coronary Artery Calcification

Examining coronary artery calcification distribution revealed significant differences between the aortic valve calcification and control groups. Total coronary calcification, including LM, LAD, LCX, and RCA, was markedly higher in the calcification group (*p* < 0.05). The severity of coronary artery calcification also varied notably, with higher rates of high (≥400 points) and medium (100–400 points) calcification in the calcification group and lower rates of low calcification (<100 points) (Table 1).

### 3.5 Coronary Artery Lesion Characteristics

Left main lesions were significantly more prevalent in the aortic valve calcification group compared to the control group (23.1% vs. 13.8%, *p* < 0.001), with no significant difference in stenosis observed in this segment.

The calcification group exhibited a higher incidence of lesions in multiple vessel branches (1, 2, and 3 lesions) compared to the control group (*p* < 0.001), indicating a broader extent of coronary involvement in patients with aortic valve calcification (Table 1).

### 3.6 Multivariate Logistic Regression Analysis of the Occurrence of Aortic Valve Calcification

A multivariate logistic regression analysis was conducted to assess determinants of aortic valve calcification.

Results highlighted specific factors as independent predictors of aortic valve calcification. Hypertension (adjusted odds ratio, AOR = 2.195, 95% CI = 1.608–2.997, *p* < 0.001), diabetes mellitus (AOR = 1.469, 95% CI = 1.061–2.034, *p* = 0.021), and eGFR (AOR = 0.977, 95% CI = 0.965–0.987, *p* < 0.001) emerged as robust predictors, elucidating their roles in the calcification process. Additionally, RDW–SD (AOR = 1.055, 95% CI = 0.965–0.987, *p* = 0.005) and RDW–CV (AOR = 1.247, 95% CI = 1.115–1.394, *p* < 0.001) demonstrated predictive significance. In addition, we found no significant interaction between RDW and hypertension and diabetes mellitus in the interaction analysis (*p* for interaction >0.05), which proved RDW might be an independent predictor (Table 2).

**Table 2.** **Multiple logistic analysis results of the RDW–SD and RDW–CV in predicting aortic valve calcification**.

Total	Adjusted odds ratio (95% CI)					*p*-value					
Hypertension	2.195 (1.608–2.997)					<0.001					
Diabetes mellitus	1.469 (1.061–2.034)					0.021					
eGFR	0.977 (0.965–0.987)					<0.001					
RDW–CV	1.247 (1.115–1.394)					<0.001					
RDW–SD	1.055 (0.965–0.987)					0.005					
Subgroup		Q1	Q2		*p * for interaction	Q3		*p* for interaction	Q4		*p* for interaction
Characteristics			Adjusted odds ratio (95% CI)	*p*-value		Adjusted odds ratio (95% CI)	*p*-value		Adjusted odds ratio (95% CI)	*p*-value	
RDW–CV											
Hypertension		Ref									
	Yes		0.988	0.951	0.381	1.135	0.240	0.303	1.250	0.001	0.310
			(0.638–1.528)			(0.919–1.403)			(1.091–1.431)		
	No		0.915	0.213		0.947	0.759		1.218	0.064	
			(0.456–1.840)			(0.668–1.342)			(0.989–1.499)		
Diabetes mellitus		Ref									
	Yes		0.925	0.681	0.663	1.153	0.439	0.595	1.293	0.035	0.418
			(0.428–2.002)			(0.804–1.655)			(1.018–1.641)		
	No		1.039	0.934		1.077	0.482		1.251	0.001	
			(0.684–1.576)			(0.876–1.324)			(1.101–1.422)		
eGFR		Ref									
	≥60		1.119	0.001	0.078	1.085	0.946	0.978	1.232	0.003	0.035
			(0.769–1.628)			(0.899–1.309)			(1.094–1.388)		
	<60		0.117	0.736		1.133	0.772		1.161	0.192	
			(0.011–1.212)			(0.560–2.292)			(0.747–1.803)		
RDW–SD											
Hypertension		Ref									
	Yes		1.383	0.150	0.548	1.289	0.026	0.705	1.394	<0.01	0.100
			(0.890–2.150)			(1.037–1.602)			(1.212–1.602)		
	No		1.247	0.568		1.109	0.588		1.316	0.021	
			(0.584–2.659)			(0.763–1.612)			(1.043–1.662)		
Diabetes mellitus		Ref									
	Yes		1.446	0.335	0.565	1.263	0.235	0.046	1.346	0.016	0.553
			(0.683–3.062)			(0.859–1.859)			(1.056–1.714)		
	No		1.277	0.277		1.214	0.076		1.359	0.029	
			(0.822–1.985)			(0.980–1.530)			(1.185–1.557)		
eGFR		Ref									
	≥60		1.273	0.222	0.252	1.167	0.116	0.761	1.279	0.001	0.701
			(0.864–1.876)			(0.963–1.415)			(1.130–1.447)		
	<60		5.455	0.148		2.882	0.060		2.759	0.089	
			(0.548–54.276)			(0.955–8.702)			(1.343–5.668)		

RDW–SD, red cell distribution width–standard deviation; RDW–CV, red cell distribution width–coefficient of variation; eGFR, estimated glomerular filtration rate; Q1, the first quartile; Q2, the second quartile; Q3, the third quartile; Q4, the fourth quartile.

### 3.7 Receiver Operating Characteristic (ROC) Curve Analysis

**Fig. 3.**
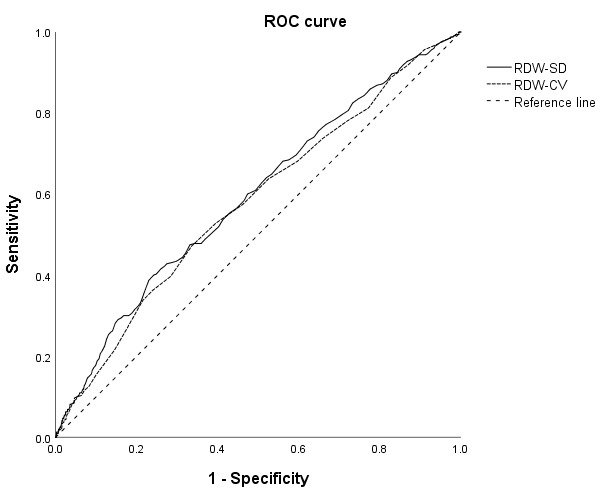
**ROC curves for RDW–SD and RDW–CV in predicting the occurrence of aortic valve calcification**. ROC, receiver operating characteristic; RDW–SD, red blood cell distribution width–standard deviation; RDW–CV, red blood cell distribution width–coefficient of variation.

The predictive capacity of RDW–SD and RDW–CV for aortic valve calcification was assessed through ROC curve analysis (Fig. 3, Table 3). These results highlighted the significant predictive value of both RDW–SD (area under the curve (AUC) = 0.594, *p* < 0.001) and RDW–CV (AUC = 0.579, *p* < 0.001). These results exhibit some statistical significance and offer a limited predictive value (Table 3).

**Table 3.** **ROC analysis results of the RDW–SD and RDW–CV in predicting aortic valve calcification**.

Characteristics	Area under curve	95% CI	*p*-value	Bounpoint (cut off point)	Sensibility	Specificity
RDW–SD	0.594	0.559–0.629	<0.001	43.8	0.399	0.770
RDW–CV	0.579	0.544–0.614	<0.001	12.9	0.472	0.664

RDW–SD, red cell distribution width–standard deviation; RDW–CV, red cell distribution width–coefficient of variation; ROC, receiver operating characteristic.

### 3.8 The Relationship between the Degree of Aortic Valve Calcification and RDW

Aortic valve calcification severity, assessed through CT double oblique transverse reconstruction, was categorized into four grades (Fig. 2): among the cohort of 1720 patients, 81.4% were classified as Grade 1, 12.9% as Grade 2, 4.2% as Grade 3, and 1.5% as Grade 4.

**Fig. 4.**
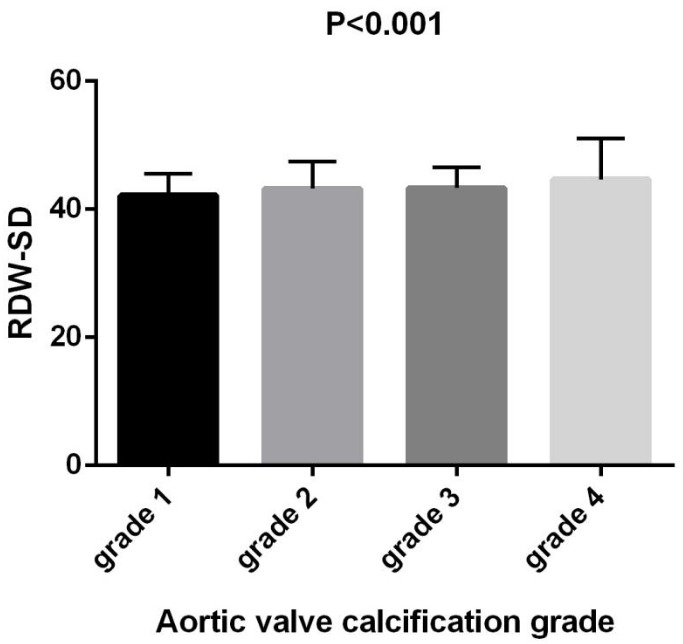
**Comparison of RDW–SD in patients with different aortic valve calcification grades**. RDW–SD, red cell distribution width–standard deviation.

**Fig. 5.**
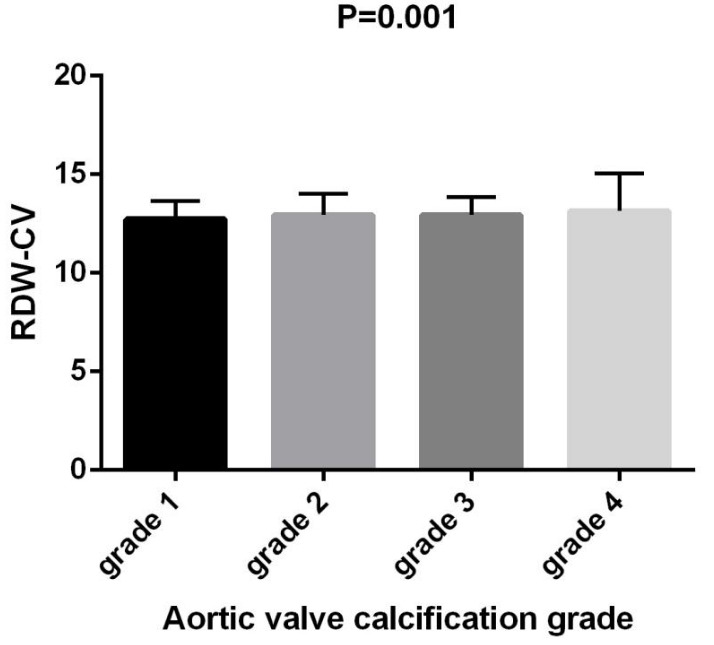
**Comparison of RDW–CV in patients with different aortic valve calcification grades**. RDW–CV, red cell distribution width–coefficient of variation.

RDW–SD values showed an increasing trend with calcification severity, ranging from 42.16 ± 3.42 in Grade 1 to 44.66 ± 6.42 mg/L in Grade 4 (F = 10.975, *p* < 0.001) (Fig. 4). Similarly, RDW–CV values exhibited an upward trajectory, from 12.72 ± 0.92 in Grade 1 to 13.15 ± 1.88 mg/L in Grade 4 (F = 5.916, *p* = 0.001) (Fig. 5).

## 4. Discussion

CAVD stands as a prevalent cardiovascular concern, following closely behind coronary heart disease and hypertension. With advancing age, the normal aortic valve undergoes a gradual process involving fibrosis, calcification, remodeling, thickening, and eventual obstruction. This progression leads to aortic valve insufficiency and stenosis, which in turn can result in syncope, myocardial infarction, heart failure, and other severe cardiovascular events. The available predictors for CAVD are currently limited, underscoring the significance of studies focusing on indicators associated with CAVD for disease prevention and treatment.

Presently, research directly linking RDW to valve calcification remains limited. However, investigations into diseases correlated with active valve calcification show promise in elucidating their relationship. Diagnosis of valve calcification relies heavily on clinical judgment and imaging examinations. Nevertheless, the predictive value of imaging in the early detection of valve-related calcification diseases still needs to be improved. Early identification using biomarkers could notably enhance disease management and prevention. Regrettably, the array of commonly used indicators in clinical practice is limited. However, RDW has gained substantial attention in recent years. Derived from a standard complete blood count (CBC), RDW assesses the size variability of circulating red blood cells, expressed as the coefficient of variation of red blood cell size. It is calculated by dividing the standard deviation (SD) of red blood cell volume by the average red blood cell volume (MCV) (i.e., RDW = SD/MCV) [14].

In cardiovascular disease, the persistent elevation of red blood cell distribution width (RDW) is attributed to the efficient stimulation of erythropoiesis by erythropoietin (EPO), a hormone secreted in response to hypoxic conditions, which initiates the proliferation and release of mature erythroid cells from the bone marrow. An alternative hypothesis posits that the heightened RDW may result from a slight decrease in red blood cell turnover, allowing smaller cells to persist in circulation for an extended period [15].

Our study disclosed significantly elevated RDW–SD and RDW–CV values in the aortic valve calcification group compared to the control group. Moreover, RDW–SD and RDW–CV demonstrated an increasing trend with escalating calcification levels. These findings underscore the predictive value of RDW–SD and RDW–CV for aortic valve calcification, corroborating existing research on RDW.

Numerous studies have robustly linked RDW to cardiovascular disease. For example, Felker *et al*. [16] investigated 2679 patients with chronic heart failure, utilizing a Cox proportional hazards model to evaluate the relationship between routine blood tests and outcomes. Their results indicated that increased RDW independently predicted morbidity and mortality in chronic heart failure patients, providing initial evidence for the prognostic value of RDW in heart failure [17]. Chronic heart failure is perceived as a systemic disease rooted in chronic inflammatory conditions characterized by a notably high mortality rate. In our study, red blood cell distribution width exhibited a statistically significant correlation with aortic valve calcification, which was in line with predictions related to heart failure. This alignment may be attributed to shared pathogenesis between heart failure and valve calcification, including inflammatory stimulation and metabolic disorders, which could impede EPO secretion and red blood cell maturation, leading to elevated RDW.

Recent research has underscored a close association between RDW abnormalities and the occurrence of atrial fibrillation (AF). The onset of AF is considered to be linked to an elevated risk of mortality due to adverse events in patients with myocardial infarction and aortic stenosis. Adamsson Eryd *et al*. [18] conducted a study monitoring 27,124 healthy individuals without cardiovascular disease (aged 45–73 years, 62% women) over an average of 13.6 years. Their findings revealed a gradual increase in the incidence of AF across RDW quartiles. A subsequent case–control study further supported these observations, demonstrating significantly higher RDW in 60 controls without AF than 117 controls (*p* < 0.05). RDW emerged as an independent predictor of AF, with age and atherosclerosis identified as additional risk factors.

A notable association exists between valve calcification and atrial fibrillation. Valve calcification, a structural change in the heart, influences the direction and speed of blood flow, thereby impacting the electrophysiological activity of the heart and leading to arrhythmia symptoms such as atrial fibrillation [18]. Yang Long *et al*. [19] proposed that activating transforming growth factor-β1 (TGF-β1)/c-Jun N-terminal kinase (JNK)–mitogen-activated protein kinase (MAPK) and extracellular regulated protein kinases (ERK)–MAPK signaling pathways might be implicated in developing atrial fibrillation secondary to valvular heart disease. This activation is also associated with reduced levels of CD4+/CD8+ T lymphocytes in local blood. The present study adds to this body of knowledge by demonstrating a significant elevation in RDW in patients with aortic valve calcification, akin to patients with atrial fibrillation. These findings contribute valuable clinical data to the exploration of how valvular heart disease may stimulate atrial fibrillation.

Furthermore, RDW, identified as a novel biomarker for chronic kidney disease (CKD), provides valuable insights into the mechanistic foundations of its heightened levels in patients with aortic valve calcification. CKD is marked by heightened oxidative stress and inflammation [20]. Furthermore, recent investigations by Kalay *et al*. [21] suggest that serum uric acid levels and RDW independently predict slow coronary flow in CKD patients. Anemia is common in CKD patients, primarily due to diminished erythropoietin production, inadequate hematopoietic raw materials, and a metabolically disordered internal environment unfavorable for red blood cell growth. The compensatory surge in blood flow, aimed at meeting the body’s oxygen consumption in CKD patients, accentuates mechanical stress changes. This phenomenon represents one of the mechanisms underlying aortic valve calcification, potentially culminating in valve calcification. Studies have shown that RDW may play a role in aortic valve calcification by inducing inflammation and affecting metabolism. At the same time, RDW has a good predictive effect on many diseases. Therefore, targeted therapy and drug research for RDW may become a new research hotspot.

Meanwhile, this study has some limitations. First, the studied population was mainly adult Chinese patients, with no multi-ethnic and multi-country studies. Second, this is a cross-sectional study, meaning it did not investigate the long-term effect of the RDW index on AVC. Third, although statistical significance was observed, the differences between groups in Figs. 3,4 are not substantial. Fourth, despite our rigorous experimental design to reduce possible errors in the study, the inherent bias in the study cannot be excluded.

## 5. Conclusions

In conclusion, upon statistical analysis of blood examinations and coronary CT results in both calcified and non-calcified groups, our findings indicate a noteworthy elevation in RDW–SD and RDW–CV values among patients in the aortic valve calcification group compared to the control group. Moreover, there is a discernible correlation between the degree of calcification and the increasing levels of RDW–SD and RDW–CV. Thus, we assert that RDW–SD and RDW–CV values are predictive indicators for aortic valve calcification.

## Availability of Data and Materials

The datasets used and/or analyzed during the current study are available from the corresponding author on reasonable request.
